# Draft Genome Sequence of a Taxonomically Unique Acinetobacter Clinical Strain with Proteolytic and Hemolytic Activities

**DOI:** 10.1128/genomeA.00030-15

**Published:** 2015-03-05

**Authors:** German Matías Traglia, Marisa Almuzara, Claudia Barberis, Sabrina Montaña, Sareda T. J. Schramm, Brandi Enriquez, María Alejandra Mussi, Carlos Vay, Andres Iriarte, María Soledad Ramírez

**Affiliations:** a Instituto de Microbiología y Parasitología Médica (IMPaM, UBA-CONICET), Buenos Aires, Argentina; b Laboratorio de Bacteriología Clínica, Departamento de Bioquímica Clínica, Hospital de Clínicas José de San Martín, Facultad de Farmacia y Bioquímica, Buenos Aires, Argentina; c Centro de Estudios Fotosintéticos y Bioquímicos (CEFOBI- CONICET), Rosario, Argentina; d Departamento de Desarrollo Biotecnológico, Facultad de Medicina, Instituto de Higiene, Montevideo, Uruguay; e Departamento de Bioquímica y Genómica Microbianas and Departamento Genómica, IIBCE, Montevideo, Uruguay; f Department of Biological Science, Center for Applied Biotechnology Studies, California State University Fullerton, Fullerton, California, USA

## Abstract

Acinetobacter sp. strain A47, which has been recovered from several soft tissue samples from a patient undergoing reconstructive surgery due to a traumatic amputation, was categorized as a taxonomically unique bacterial strain. The molecular analysis based on three housekeeping protein-coding genes (16S rRNA, *rpoB*, and *gyrB*) showed that strain A47 does not belong to any of the hitherto known taxa and may represent a previously undescribed Acinetobacter species.

## GENOME ANNOUNCEMENT

Acinetobacter is a complex genus that currently comprises at least thirty-three distinct Acinetobacter species with valid names (http://www.bacterio.net/acinetobacter.html), as well as several provisionally termed genomic species (1) or unique strains that do not belong to any of these thirty-three species (2–4). Molecular methods are required to determine the correct identification of the Acinetobacter species, and the correct identification is still a challenge, not only for conventional microbiology laboratories, but also for reference laboratories due to the taxonomic complexity of the genus (2).

Here, we report the draft genome sequence of a taxonomically unique Acinetobacter sp. strain (A47). The strain was isolated from several soft tissue samples from a 59-year-old female patient with a history of chronic alcoholism, who was admitted to the emergency room due to severe left forearm trauma and traumatic amputation of her left foot secondary to a road accident (5).

Different phenotypic and molecular methods were used to identify correctly the present strain, demonstrating that A47 does not belong to any of the hitherto known taxa and may represent a currently undescribed Acinetobacter species (5). Based on the *rpoB* and *gyrB* sequences this strain appeared to be most closely related to Acinetobacter species that typically include hemolytic and/or proteolytic strains, such as Acinetobacter gyllenbergii, Acinetobacter venetianus, or genomic species described by Bouvet and Jeanjean (6).

The draft genome of this taxonomically unique Acinetobacter sp. A47 strain was obtained using Illumina MiSeq at the Argentinian Consortium of Genomic Technology (ACGT). A total of 1,705,036 high-quality paired-end reads were produced with an average insertion size of 485. *De novo* assembly was performed with SPAdes assembler version 3.1.0 (7), using a preassembly approach with Velvet (8); 1,687,796 paired reads plus 7,498 unpaired reads (99.4% of the generated reads) were assembled, resulting in a mean nucleotide coverage of 73 (and a *k*-mer coverage of 23). Corrected reads showed an average length of 171 bp. The assembled contigs totaled 3,915,593 bp with an *N*_50_ of 511,473 (max length 661,192) and a G+C content of 44.5%. The RAST server was used to predict and annotate the open reading frames (ORFs) present in A47, showing the presence of 3,627 possible ORFs. Using the tRNAscan-SE, 73 tRNA genes were identified (9).

The complete genome sequence of the A47 strain opens up a wide path to explore in detail the metabolic and physiological properties of this taxonomically unique strain and shines a light on the understanding of the taxonomic complexity underlying infections caused by Acinetobacter. Moreover, it will allow us to perform comparative sequence studies and phylogenetic analyses with other Acinetobacter species, further expanding our understanding of this complex genus. A complete characterization of relevant traits present in this strain, as well as whole-genome sequence comparison studies, will be included in a future publication.

### Nucleotide sequence accession numbers.

This whole-genome shotgun project has been deposited at DDBJ/EMBL/GenBank under the accession number JSUS00000000. The version described in this paper is version JSUS01000000.
